# Fur Represses *Vibrio cholerae* Biofilm Formation *via* Direct Regulation of *vieSAB*, *cdgD*, *vpsU*, and *vpsA-K* Transcription

**DOI:** 10.3389/fmicb.2020.587159

**Published:** 2020-10-22

**Authors:** He Gao, Lizhi Ma, Qin Qin, Yue Qiu, Jingyun Zhang, Jie Li, Jing Lou, Baowei Diao, Hongqun Zhao, Qiannan Shi, Yiquan Zhang, Biao Kan

**Affiliations:** ^1^State Key Laboratory of Infectious Disease Prevention and Control, National Institute for Communicable Disease Control and Prevention, Chinese Center for Disease Control and Prevention, Beijing, China; ^2^Third Medical Center of Chinese PLA General Hospital, Beijing, China; ^3^School of Medicine, Jiangsu University, Zhenjiang, China; ^4^Wuxi School of Medicine, Jiangnan University, Wuxi, China

**Keywords:** *Vibrio cholerae*, biofilm, *vps*, c-di-GMP, Fur

## Abstract

Attached *Vibrio cholerae* biofilms are essential for environmental persistence and infectivity. The *vps* loci (*vpsU*, *vpsA*-K, and *vpsL-Q*) are required for mature biofilm formation and are responsible for the synthesis of exopolysaccharide. Transcription of *vps* genes is activated by the signaling molecule bis-(3′–5′)-cyclic di-GMP (c-di-GMP), whose metabolism is controlled by the proteins containing the GGDEF and/or EAL domains. The ferric uptake regulator (Fur) plays key roles in the transcription of many genes involved in iron metabolism and non-iron functions. However, roles for Fur in *Vibrio* biofilm production have not been documented. In this study, phenotypic assays demonstrated that Fur, independent of iron, decreases *in vivo* c-di-GMP levels and inhibits *in vitro* biofilm formation by *Vibrio cholerae*. The Fur box-like sequences were detected within the promoter-proximal DNA regions of *vpsU*, *vpsA-K*, *vieSAB*, and *cdgD*, suggesting that transcription of these genes may be under the direct control of Fur. Indeed, the results of luminescence, quantitative PCR (qPCR), electrophoretic mobility shift assay (EMSA), and DNase I footprinting assays demonstrated Fur to bind to the promoter-proximal DNA regions of *vpsU*, *vpsA-K*, and *cdgD* to repress their transcription. In contrast, Fur activates the transcription of *vieSAB* in a direct manner. The *cdgD* and *vieSAB* encode proteins with GGDEF and EAL domains, respectively. Thus, data presented here highlight a new physiological role for Fur wherein it acts as a repressor of *V. cholerae* biofilm formation mediated by decreasing the production of exopolysaccharide and the intracellular levels of c-di-GMP.

## Introduction

*Vibrio cholerae*, an inhabitant of aquatic ecosystems, is the causative agent of the fatal waterborne diarrheal disease cholera (Clemens et al., 2017). *V. cholerae* is most often found in the form of biofilm communities attached to biotic and abiotic surfaces (Yildiz and Visick, 2009). These biofilms have been proven essential for the environmental persistence and infectivity of *V. cholerae* (Faruque et al., 2006; Tamayo et al., 2010; Sengupta et al., 2016). During infection, the expression of biofilm-related genes is upregulated within a short time, and this accounts for the immediate initiation of biofilm formation after adherence of *V. cholerae* to intestinal cells, and thus, biofilm-like aggregates of *V. cholerae* are frequently shed in the stool of cholera patients (Faruque et al., 2006; Tamayo et al., 2010; Sengupta et al., 2016). The ability of *V. cholerae* to form biofilms *in vivo* is critical for its survival in humans and for the transmission of cholera (Tamayo et al., 2010). Formation of mature biofilms requires specific extracellular matrix components, among which the exopolysaccharide is the most significant one (Yildiz and Visick, 2009). *V. cholerae* exopolysaccharide (VPS) is encoded by *vps* loci, which are clustered in two regions, the *vps*-I cluster (*vpsU* and *vpsA*-K) and the *vps*-II cluster (*vpsL-Q*) (Fong et al., 2010). The two regions are separated by the *rbm* gene cluster (Fong et al., 2010). Deletion of any of the *vps* genes produces smooth colony morphology with decreased biofilm formation *in vitro* (Fong et al., 2010, 2017).

The second messenger bis-(3′–5′)-cyclic di-GMP (c-di-GMP) promotes biofilm formation and inhibits bacterial motility (Mills et al., 2011). *V. cholerae* responds to an elevated level of c-di-GMP by increasing the transcription of *vps*, *eps*, and *msh* genes and by decreasing flagellar gene transcription (Beyhan et al., 2006). c-di-GMP is synthesized from two molecules of GTP by diguanylate cyclases (DGCs) containing a GGDEF motif. It is degraded into 5′-phosphoguanylyl-(3′–5′)-guanosine (pGpG) and/or GMP by phosphodiesterases (PDEs) carrying either EAL or HD-GYP domains (Valentini and Filloux, 2016). A set of genes encoding a group of proteins that synthesize and/or degrade c-di-GMP has been identified in *V. cholerae*, including *acgAB* (Kovacikova et al., 2005; Pratt et al., 2009), *cdgA* [(Beyhan et al., 2007; Fong and Yildiz, 2008), *cdgC* (Lim et al., 2007), *cdgD* (Syed et al., 2009), *cdgG* (Beyhan et al., 2008; Fong and Yildiz, 2008), *cdgH* [(Beyhan et al., 2008; Fong and Yildiz, 2008), *cdgI* (Fong and Yildiz, 2008), *vieSAB* (Tamayo et al., 2005; Dey et al., 2013), and *vvpC* (Beyhan and Yildiz, 2007). Mutation of any of these genes alters VPS production and biofilm formation. c-di-GMP regulates gene expression by binding transcriptional regulators, such as VpsR and VpsT, both of which can bind to *vpsL-Q* promoters to positively regulate their transcription (Beyhan et al., 2007; Srivastava et al., 2011; Shikuma et al., 2012; Zamorano-Sanchez et al., 2015). A number of other transcriptional regulators have been identified that impact the transcription of *vps* genes and *V. cholerae* biofilm formation *via* control of the expression of *vpsR* and/or *vpsT*. These include the positive regulator VxrB (Teschler et al., 2017) and the negative regulators NtrC (Cheng et al., 2018), H-NS (Wang et al., 2012; Ayala et al., 2015), cAMP-CRP complex (Fong and Yildiz, 2008), CarR (Bilecen and Yildiz, 2009), and PhoB (Sultan et al., 2010). VpsT and VpsR also function as anti-repressors of H-NS repression in the presence of c-di-GMP (Wang et al., 2012; Ayala et al., 2015). Quorum sensing (QS) regulates *vps* genes primarily *via* the master regulators, AphA and HapR (Ng and Bassler, 2009). At low cell density, AphA is highly expressed and thus promotes biofilm formation through direct activation of *vpsT* (Yang et al., 2010). At high cell density, highly expressed HapR binds to and directly represses the expression of *vpsT* (Zhu and Mekalanos, 2003; Waters et al., 2008). Thus, transcription of the *vps* genes and biofilm formation are regulated by a complex regulatory network that involves c-di-GMP, multiple transcriptional regulators, and QS. Although much has been learned about the regulation of *V. cholerae* biofilm formation, a full understanding of this complex process has not been elucidated.

The ferric uptake regulator (Fur) is a typically iron-dependent DNA-binding protein that controls a group of genes involved in iron homeostasis, virulence, ribosome formation, transporters, and unique sRNAs (Occhino et al., 1998; Davis et al., 2005; Mey et al., 2005; Wyckoff et al., 2007; Davies et al., 2011). Fe^2+^-Fur regulates transcription of target genes by recognizing and binding to conserved Fur boxes within promoter regions. The Fur box in *V. cholerae* has been described as a 19-bp palindromic sequence, i.e., AATGATAATNATTATCATT (Goldberg et al., 1990). An enhanced *V. cholerae* Fur box was reconstructed as a 21-bp palindromic sequence based on ChIP-seq-identified binding sites (Davies et al., 2011). This construct shares an identical span of bases described for the original sequence (Davies et al., 2011).

Fur regulation of biofilm formation has been reported in other species (Banin et al., 2005; Johnson et al., 2005; Sun et al., 2012; Wu et al., 2012; Liu et al., 2016; Pasqua et al., 2017; Tanui et al., 2017) but not in Vibrios. In the present work, we investigated the regulation of biofilms by iron and Fur in *V. cholerae*. The results showed that Fur represses the biofilm formation of *V. cholerae*, at least in part, by regulating the transcription of *cdgD*, *vieSAB*, *vpsU*, and *vpsA-K*, in an iron-independent manner. Thus, we identified a new physiological role for Fur in *V. cholerae*.

## Materials and Methods

### Mutation of *fur* and Complementation of the Mutant

*Vibrio cholerae* strain C7258 (El Tor; Peru, 1991) was used as the wild type (WT). The *fur* mutant (designated as Δ*fur*) was constructed from WT using the suicide plasmid pWM91 by allelic exchange, as previously described (Wu et al., 2015). The coding region of *fur* was cloned into the pBAD24 harboring an arabinose PBAD promoter and an ampicillin resistance gene. After being verified by DNA sequencing, the recombinant pBAD24 plasmid was introduced into the Δ*fur* strain yielding the complementary mutant strain Δ*fur*/pBAD24-*fur* (Guzman et al., 1995; Sun et al., 2014). The empty pBAD24 was also introduced into WT and Δ*fur* to generate WT/pBAD24 and Δ*fur*/pBAD24 to counteract the effects of arabinose and ampicillin on bacterial growth (Sun et al., 2014). All the primers used were listed in Supplementary Table 1.

### Growth Conditions

*Vibrio cholerae* strains were routinely cultured in Luria-Bertani (LB) broth (1% tryptone, 0.5% yeast extract, and 1% NaCl). The bacterial cells were grown overnight at 30°C with shaking at 200 rpm. The bacterial cultures were diluted 1:50 into 15 ml of fresh LB broth and grown to an OD_600_ value of 1.0. The resultant cultures were then diluted 1:100 into 5 ml of fresh LB broth for a third-round growth. For the iron-starved condition, the medium used for the third-round culture was treated with 100 μM iron chelator 2, 2′-dipyridyl (DP). For the iron-replete condition, ferrous sulfate (FeSO_4_) was added to a final concentration of 40 μM. The cultures were harvested at an OD_600_ value of approximately 0.6. When appropriate, the LB broth was supplemented with 100 μg/ml ampicillin, 5 μg/ml chloramphenicol, 50 μg/ml kanamycin, or 0.1% arabinose. *V. cholerae* is a biosafety level 2 (BSL-2) pathogen, and thus all experimental procedures involving live bacteria were completed in a BSL-2 lab.

### Colony Morphology

The rugose colony morphology assay was performed as previously described (Fong et al., 2010; Fang et al., 2013). Briefly, *V. cholerae* strains were cultivated overnight in LB broth, and 2 μl of each culture was spotted onto LB agar, LB agar supplemented with 100 μM DP, and LB agar supplemented with 40 μM FeSO_4_, respectively. The plates were statically incubated at 30°C for about 2 days.

### Crystal Violet Staining

The crystal violet (CV) staining assay was performed as previously described (Fang et al., 2013). Briefly, the third-round of cultivated bacterial cells was 50-fold diluted, respectively, into 2 ml of fresh LB broth, LB supplemented with 100 μM DP, and LB supplemented with 40 μM FeSO_4_ in a 5-ml glass tube and allowed to grow at 30°C with shaking at 100 rpm for 48 h. The surface-attached cells (the adherent biofilms) were stained with 0.1% CV. The bound dye was dissolved with dimethylsulfoxide (DMSO), and OD_570_ values were determined as an index of CV staining.

### Scanning Electron Microscopy

Scanning Electron Microscopy (SEM) was performed as previously described (Tan et al., 2018). Briefly, after 48 h of incubation at 30°C with shaking at 100 rpm, the biofilms on slides were fixed for 12 h at 4°C in a solution containing 4% glutaraldehyde. The slides were then washed three times with 0.1 M phosphate buffered saline (PBS). The slides were dehydrated in serial dilutions of 30, 50, 60, 70, 90, and 95% ethanol for 10 min each, followed by two 10-min rinses in 100% ethanol. The slides were dried and coated with gold-palladium in an automatic sputter coater (Hitachi Ion Sputter MC1000, Japan). Biofilms were visualized by using a scanning electron microscope (FESEM HITACHI SU8010, Japan). The images were acquired for three independent replicates.

### Extraction and Quantification of c-di-GMP

The intracellular c-di-GMP concentration was measured as previously described (Meng et al., 2020). Briefly, the third-round of cultivated bacterial cells was 50-fold diluted, respectively, into 2 ml of fresh LB broth, LB supplemented with 100 μM DP, and LB supplemented with 40 μM FeSO_4_ in a 5-ml glass tube and allowed to grow at 30°C with shaking at 200 rpm to an OD_600_ = 0.6∼0.8. Two milliliters of planktonic culture was centrifuged at 10,000 × *g* for 5 min at 4°C. The cell pellet was washed twice with ice-cold PBS, resuspended in 2 ml ice-cold PBS, incubated at 100°C for 5 min, and sonicated for 15 min (power 100%, frequency 37 kHz) in an ice-water bath. After centrifugation, the supernatant containing extracted c-di-GMP was collected, and the pellet was resuspended in 2 ml ice-cold PBS. The pellet was re-extracted another two times. The extracts were concentrated by cooling evaporation at 4°C to a volume of 500 μl, and intracellular c-di-GMP levels were determined with a c-di-GMP enzyme-linked immunosorbent assay (ELISA) kit (Mskbio, Beijing, China). Cell protein was determined by the bicinchoninic acid (BCA) assay, and c-di-GMP concentrations were expressed as pmol/mg of protein.

### Luminescence Assay

The luminescence assay was performed as previously described (Xu et al., 2010). Briefly, the promoter DNA region of each target gene was cloned into a pBBRlux vector harboring a promoterless *luxCDABE* reporter gene and a chloramphenicol resistance gene. The resultant plasmid was transferred into WT and Δ*fur* to measure *lux* activity in the strains. Luminescence was measured using an Infinite^®^ 200 Pro NanoQuant (Tecan, Switzerland). The *lux* activity was calculated as light units/OD_600_.

### Quantitative PCR

The quantitative PCR (qPCR) assay was performed as previously described (Gao et al., 2011). Briefly, total RNA was extracted from WT and Δ*fur* using TRIzol reagent (Invitrogen, United States). The cDNAs were generated using 8 μg of total RNA and 3 μg of random hexamer primers. The relative mRNA levels were determined based on a standard curve of *recA* (reference gene) expression for each RNA preparation.

### Preparation of 6× His-Tagged Fur Protein

The entire coding region of *fur* was cloned into plasmid pET28a (Novagen, United States) and then transferred into *Escherichia coli* BL21λDE3 cells for His-tagged Fur protein (His-Fur) expression (Kleber-Janke and Becker, 2000). Purification of overexpressed His-Fur was performed as previously described (Gao et al., 2008). The eluted His-Fur was dialyzed with the storage buffer containing Tris–HCl (pH 7.5), 50 mM NaCl, 2 mM CaCl_2_, and 15% glycerol. The dialyzed His-Fur was concentrated using polyethylene glycol 2000 to a final concentration of 0.3 to 0.6 mg/ml. The purity of the resulting His-Fur was analyzed by sodium dodecyl sulfate (SDS)-10% polyacrylamide gel electrophoresis (PAGE).

### Electrophoretic Mobility Shift Assay

Electrophoretic mobility shift assay (EMSA) was performed as previously described (Zhang L. et al., 2017). Briefly, the entire upstream promoter-proximal DNA region of each target gene was amplified by PCR. DNA binding was performed in a 10-μl reaction volume containing binding buffer [1 mM MgCl_2_, 100 μM MnCl, 0.5 mM dithioreitol (DTT), 50 mM KCl, 10 mM Tris–HCl (pH 7.5), 0.05 mg/ml bovine serum albumin (BSA), and 10 mg/ml salmon sperm DNA], 100 ng target promoter-proximal DNA, and increasing amounts of His-Fur. After incubation at room temperature for 20 min, the products were loaded onto a native 6% (w/v) polyacrylamide gel and electrophoresed in 1.0× TB buffer at 120 volts. After staining with GelRed dye, the gel was imaged by a UV transilluminator.

### DNase I Footprinting

DNase I footprinting assays were performed as previously described (Gao et al., 2017; Osei-Adjei et al., 2017; Zhang Y. et al., 2017). Briefly, the FAM (or HEX)-labeled DNA probes were incubated with increasing amounts of His-Fur in a final 10 μl reaction volume containing the binding buffer the same as EMSA. The reaction mixture was incubated at room temperature for 30 min, then 10 μl of a Ca^2+^/Mg^2+^ solution (5 mM CaCl_2_ and 10 mM MgCl_2_) was added, followed by incubation for another 1 min at room temperature. Optimized RQ1 RNase-Free DNase I (Promega) was added to the reaction mixture and incubated at room temperature for 30 to 50 s to digest DNA fragments. Then, 9 μl of a stop solution [200 mM NaCl, 30 mM ethylenediaminetetraacetic acid (EDTA), and 1% SDS] was added to the reaction mixture to stop digestion. Digested DNA probes were extracted with a Beaver Beads^TM^ PCR Purification Kit (Beaver). BigDye^®^ Terminator v3.1 Cycle Sequencing Kits (ABI) were used for DNA sequencing. The digested DNA probes were analyzed using an ABI 3500XL DNA Genetic analyzer with GeneMarker software 2.2. DNA sequencing products were analyzed with Sequence Scanner software v1.0.

### Primer Extension Assay

The primer extension assay was performed as previously described (Gao et al., 2011, 2018). Briefly, approximately 10 μg of total RNA was annealed with 1 pmol of 5′-HEX-labeled reverse primer to generate cDNAs using a Primer Extension System (Promega) according to the manufacturer’s instructions. The primer extension products and sequencing materials were extracted and analyzed by DNase I footprinting methods.

### Experimental Replicates and Statistical Methods

Colony morphology, CV staining, EMSA, and DNase I footprinting measurements were the result of at least three independent experiments with similar results. The measurements for c-di-GMP concentration, luminescence assay, and qPCR were of at least three independent bacterial cultures, with values expressed as the means ± standard deviation (SD). A two-way ANOVA with Tukey’s *post hoc* corrections for multiple comparisons was used to calculate statistical significance. *P* < 0.01 was considered significant.

## Results

### Fur Represses Biofilm Formation in an Iron-Independent Manner

**FIGURE 1:**
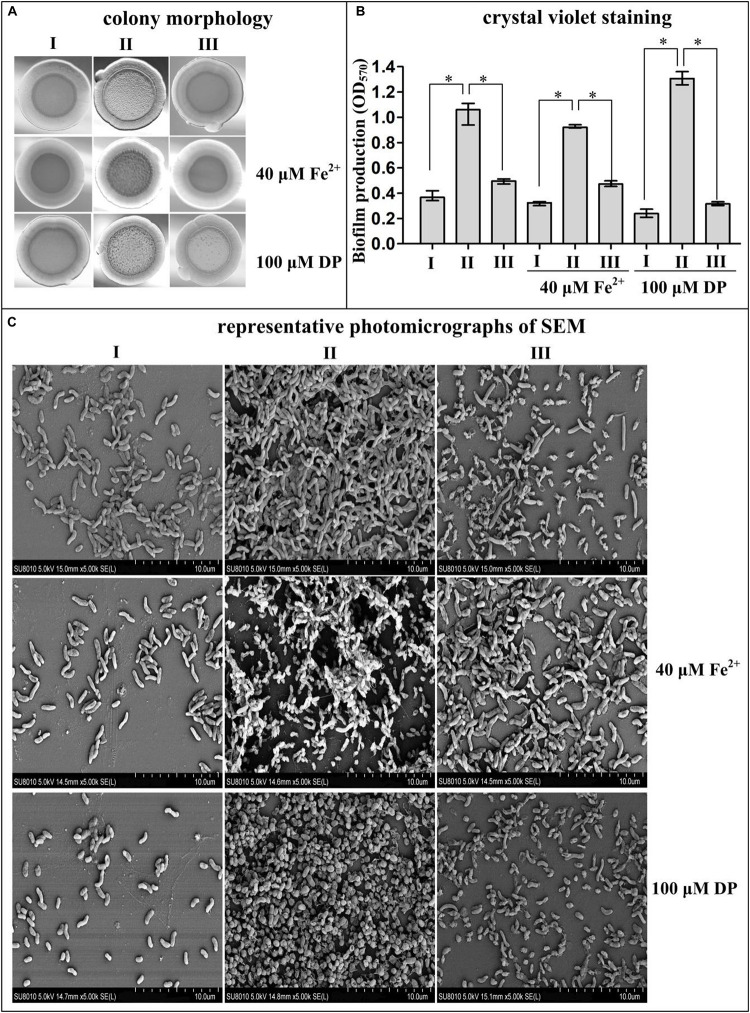
Fur represses biofilm formation by *Vibrio cholerae*. Biofilm EPS production and biofilm adherence to abiotic surfaces were measured by rugose colony morphology **(A)**, intensity of crystal violet staining **(B)**, and photomicrographs of SEM **(C)**. I, II, and III represent WT/pBAD24, Δ*fur*/pBAD24, and Δ*fur*/pBAD24-*fur*, respectively. Pictures presented in panels **(A,C)** were representative of three independent experiments with three replicates each. The asterisk (*) represents *P* < 0.01, which was determined by a two-way ANOVA with Tukey’s *post hoc*.

To characterize the biofilm formation regulated by iron and Fur in *V. cholerae* strain C7258 (El Tor), several biofilm phenotype assays were performed, which compared biofilm morphology and quantity in the Δ*fur* and WT strains grown in normal, iron-replete, and iron-starved conditions. As shown in Figure 1A, Δ*fur*/pBAD24 produced rugose colonies with each tested growth condition, while WT/pBAD24 formed smooth colonies. Δ*fur*/pBAD24-*fur* had similar colony morphology to that of WT/pBAD24. Surface-attached biomass (*in vitro* biofilms) of *V. cholerae* was assessed by the CV staining assay. As shown in Figure 1B, for each tested growth condition, Δ*fur*/pBAD24 had significantly greater normalized CV staining than WT/pBAD24 (*P* < 0.01), whereas Δ*fur*/pBAD24-*fur* produced similar CV staining to the WT/pBAD24 strain. Finally, the structure of the *V. cholerae* biofilm was examined by SEM. As shown in Figure 1C, the biofilms formed by WT/pBAD24 for all tested conditions consisted mostly of single cells with no obvious aggregations. Biomass formed by Δ*fur*/pBAD24 adhered closely, suggesting that large aggregates of cells were formed. In addition, aggregations of Δ*fur*/pBAD24-*fur* biofilms were not common and were a mass of individual cells. The morphology of the individual cells grown with DP supplementation was obviously changed. In particular, Δ*fur*/pBAD24 changed into an approximate spherical shape. Taken together, these results suggested that deletion of *fur* results in enhanced biofilm formation independent of the presence or lack of Fe^2+^.

### Deletion of *fur* Increases the Intracellular c-di-GMP Concentration

**FIGURE 2:**
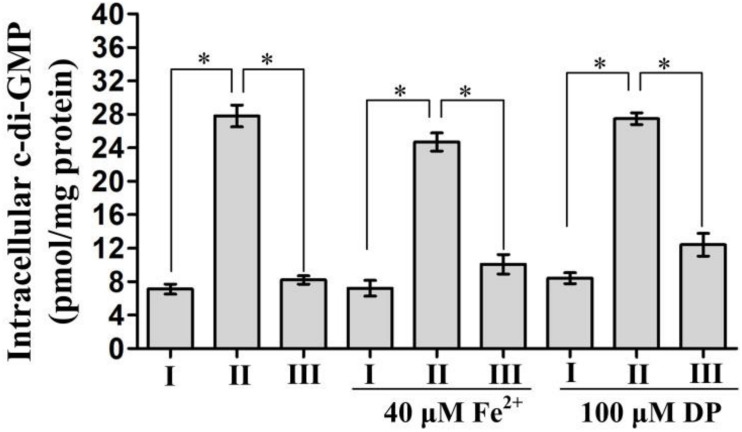
Intracellular c-di-GMP concentrations in *Vibrio cholerae* strains. Bacterial cells were cultured in Luria-Bertani (LB) broth, LB supplemented with 100 μM 2′-dipyridyl (DP), and LB supplemented with 40 μM FeSO_4_ at 30°C. Intracellular c-di-GMP concentrations were analyzed in bacteria grown to an OD_600_ = 0.6∼0.8. The data are expressed as the mean ± SD of at least three independent experiments. I, II, and III represent WT/pBAD24, Δ*fur*/pBAD24, and Δ*fur*/pBAD24-*fur*, respectively. The symbol ^∗^ represents the significant difference with *P* < 0.01.

In *Yersinia pestis*, Fur has been shown to be involved in the inhibition of c-di-GMP production (Sun et al., 2012). Thus, we assessed whether Fur regulated c-di-GMP production by Fur in *V. cholerae*. As shown in Figure 2, a significantly enhanced intracellular c-di-GMP concentration was observed for Δ*fur*/pBAD24 for each tested growth condition when compared to that for WT/pBAD24 and Δ*fur*/pBAD24-*fur*. However, there was no significantly different production of c-di-GMP between WT/pBAD24 and Δ*fur*/pBAD24-*fur* for all growth conditions. These results suggested that Fur inhibits the synthesis of c-di-GMP in *V. cholerae* independent of the concentration of Fe^2+^.

### Fur Activates *vieSAB* but Represses *cdgD* and *vps* Transcription

**FIGURE 3:**
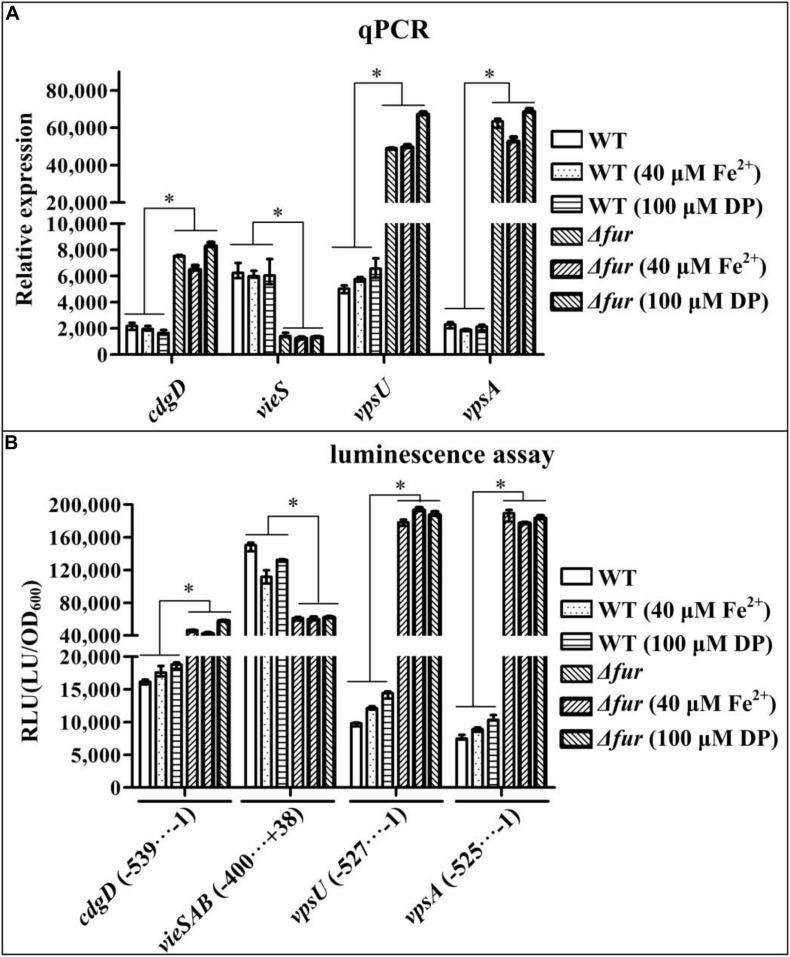
Fur regulates the expression of *vieSAB*, *cdgD*, *vpsA*, and *vpsU*. Bacterial cells were harvested at an OD_600_ value of approximately 0.6. Statistical differences between wild type (WT) and Δ*fur* (^∗^ at *P* < 0.01) were determined by a two-way ANOVA with Tukey’s *post hoc*. **(A)** Quantitative PCR (qPCR) assay was employed to determine relative mRNA levels for each target gene in Δ*fur* and WT using a standard curve of *recA* (reference gene) expression for each RNA preparation. **(B)** For the luminescence assay, the entire promoter DNA region of each target gene was cloned into a pBBRlux vector, and then introduced into Δ*fur* and WT, to determine the luminescence activity for each strain using an Infinite^®^ 200 Pro NanoQuant. The *lux* activity (RLU) was calculated as light units/OD_600_. The minus and positive numbers represented the nucleotide positions upstream and downstream of the translation start site of each target gene.

Four biofilm-related genes *vieS* (the first gene of the *vieSAB* operon, c-di-GMP degradation/phosphodiesterase), *cdgD* (c-di-GMP production/guanylate cyclase), *vpsA* (the first gene of the *vpsA-K* operon; exopolysaccharide production), and *vpsU* (exopolysaccharide production) were selected as target genes. qPCR was performed to determine Fur-dependent transcriptional changes in these biofilm-related genes. The bacterial cells were harvested at an OD_600_ value of approximately 0.6 in that expression of Fur was the highest at this cell density (Supplementary Figure 1). As shown in Figure 3A, in the conditions of normal, iron-replete, and iron-starved, the mRNA levels of *cdgD*, *vpsA*, and *vpsU* were all significantly increased in Δ*fur* relative to that of WT, while the mRNA level of *vieS* was significantly decreased in Δ*fur* (*P* < 0.01). The mRNA levels of these genes showed no obvious difference for WT or Δ*fur* for the conditions of normal, iron-replete, and iron-starved. These results suggested that *V. cholerae* Fur, independent of iron, acts as a negative regulator of *cdgD*, *vpsA-K*, and *vpsU* and as a positive regulator of *vieSAB*.

### Fur Regulates the Promoter Activities of *vieSAB*, *cdgD*, and *vps*

Recombinant pBBRlux plasmids containing the promoter DNA regions for *vieS*, *cdgD*, *vpsA*, and *vpsU* were introduced into Δ*fur* and WT to test the effect of Fur on the promoter activity of each target gene. As shown in Figure 3B, for the three growth conditions, the promoter activities of *cdgD*, *vpsA*, and *vpsU* in Δ*fur* were all significantly higher than those in WT, whereas luminescence under the control of the *vieSAB* promoter in Δ*fur* was significantly lower than that in WT (*P* < 0.01). The promoter activities of these genes showed no obvious difference in WT or in Δ*fur* for the conditions of iron-starved, normal, and iron-replete. These results suggested that expression levels of *cdgD*, *vpsA-K*, and *vpsU* were under negative control of Fur, independent of iron concentration, while *vieSAB* was positively regulated by Fur.

### His-Fur Bound to the Promoter-Proximal DNA Regions of Target Genes

**FIGURE 4:**
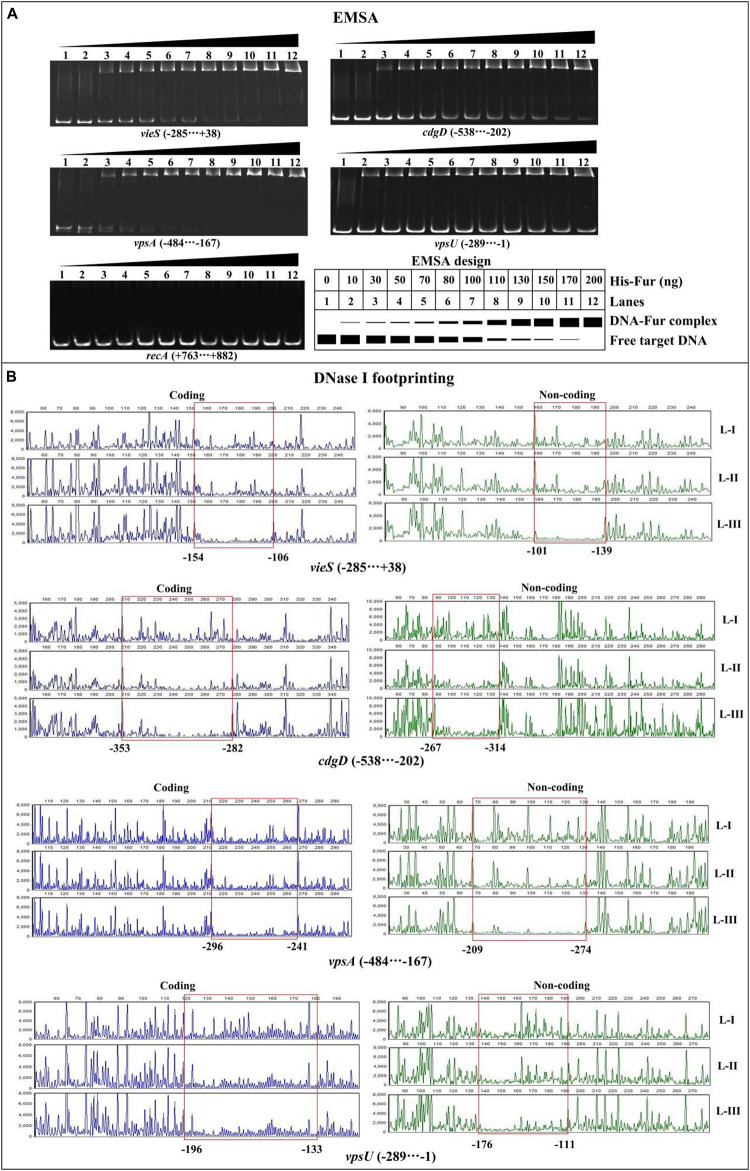
Binding of Fur to the target promoters. The negative and positive numbers indicated the nucleotide positions upstream and downstream of indicated genes, respectively. **(A)** Electrophoretic mobility shift assay (EMSA). The promoter-proximal DNA fragments of each target gene were incubated with increasing amounts of His-Fur and then subjected to 6% (w/v) polyacrylamide gel electrophoresis. The DNA bands were visualized by GelRed staining. Schematic representation of the EMSA design was shown below. **(B)** DNase I footprinting assay. Fluorescently labeled DNA probes were incubated with increasing quantities of His-Fur (Lanes-I, II, and III contain 0, 2.95, and 8.85 pmol, respectively) and digested with DNase I. Results were analyzed with an ABI 3500XL DNA analyzer. Footprint regions are boxed and marked with positions.

In order to detect whether or not Fur binds to the promoter DNA regions of the target genes, 500 bp of upstream DNA sequences for *vieS*, *cdgD*, *vpsA*, and *vpsU* were downloaded from the genome database of C7258. The DNA binding box of Fur (Zhou et al., 2006; Gao et al., 2008) was used to predict the presence of Fur box-like sequences within the target DNA fragments using the online matrix scan tool (van Helden, 2003). The analysis generated a weight score for each sequence, with higher score values indicative of a higher probability for Fur direct binding. With the weight score cutoff of 7.0 (Sun et al., 2012), Fur box-like sequences were found for the four target genes (Supplementary Table 2). Thus, the EMSA and DNase I footprinting assays were used to detect the binding of His-Fur to the regulatory regions of *vieS*, *cdgD*, *vpsA*, and *vpsU*. EMSA results showed that His-Fur bound to the promoter-proximal DNA fragment of each target gene in a dose-dependent manner in *vitro* but was unable to bind to the DNA fragment of *recA*, which was used as a negative control (Figure 4A). As further demonstrated by DNase I footprinting (Figure 4B), His-Fur protected a single region for each promoter, located from 154 to 101 bp, from 353 to 267 bp, from 296 to 209 bp, and from 196 to 111 bp upstream of the translation start site of *vieS*, *cdgD*, *vpsA*, and *vpsU*, respectively. These results demonstrated that Fur directly regulates transcription of *vieS*, *cdgD*, *vpsA*, and *vpsU*.

### Identification of the Transcription Start Sites for *cdgD*, *vpsA*, and *vpsU*

**FIGURE 5:**
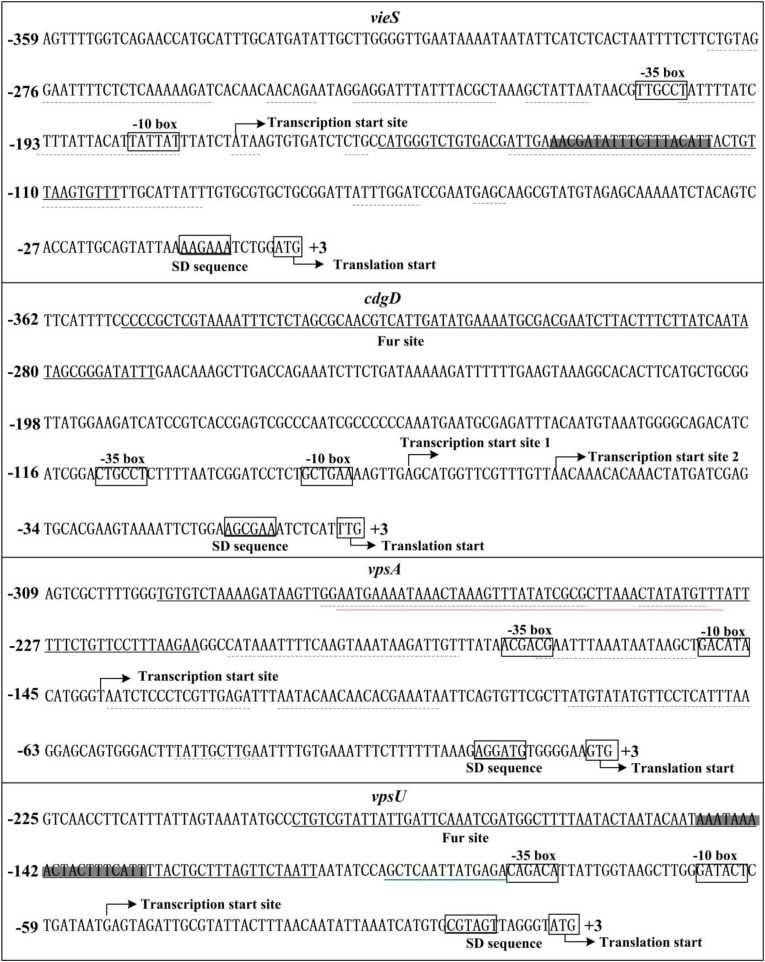
Structural organization of targeted promoters. The DNA sequences were derived from *Vibrio cholerae* El Tor C7258. Transcription/translation start sites were marked with a bent arrow. Shine-Dalgarno (SD) box and –10/–35 elements were enclosed in boxes. The Fur-box like sequences was enclosed in shadowed boxes. The Fur sites were underlined with solid lines. The H-NS sites were underlined with dotted lines (Ayala et al., 2015, 2018). The VpsT site was underlined with a red line (Ayala et al., 2015). The blue line indicated the VpsR-box-like sequence (Zamorano-Sanchez et al., 2015).

Transcription start site of *vieS* has been identified by 5′-RACE in a previous study (Ayala et al., 2018). Herein, the transcription start sites for *cdgD*, *vpsA*, and *vpsU* were determined. As shown in Supplementary Figure 2A, two transcription start sites for *cdgD* located at 74 and 57 bp upstream of the coding region, termed transcription start site 1 and 2, respectively, were detected by the primer extension assay. A non-conservative −10/−35 core promoter was detected for transcription start site 1, but no core elements were predicted for transcription start site 2 (Figure 5). Thus, transcription start site 2 was most likely a premature termination product of primer extension.

Because the primer extension assay did not map any transcription start sites for *vpsA* and *vpsU*, 5′-RACE was used to determine transcription start sites for these two genes. Total RNA was isolated from cultures of the WT strain, and an adapter tail was added to the 5′ end of the RNA, after which synthesis of cDNA was carried out using random hexamer primers. The prominent PCR product of each target gene, which was generated by nested PCR from cDNA templates, was cloned into the T easy vector and analyzed by DNA sequencing. The results showed that the transcription start sites of *vpsA* and *vpsU* were located at 139 and 52 bp upstream of the translation start sites, respectively (Supplementary Figure 2B). Each of the 5′-upstream regions of the two start sites contained the conservative −10/−35 core elements (Figure 5).

## Discussion

The regulatory effects of Fur on biofilm formation have been reported in various species. Fur has been shown to be a positive regulator of biofilm formation and exopolysaccharide production in *Pectobacterium carotovorum* subsp. *Brasiliense* and *Xanthomonas vesicatoria* (Liu et al., 2016; Tanui et al., 2017). In *Staphylococcus aureus*, biofilm formation is positively regulated by Fur in low-iron conditions but negatively regulated by iron *via* a Fur-independent mechanism (Johnson et al., 2005). In *Klebsiella pneumoniae CG43*, Fur promotes biofilm formation by induced expression of Type 3 fimbriae, mediated by MrkHI (Wu et al., 2012). However, a study in *Pseudomonas aeruginosa* showed that with low iron, a Fur mutant was able to organize into more mature biofilms than the WT strain (Banin et al., 2005). In *Y. pestis*, Fur inhibits biofilm production *in vitro* by binding to and repressing the transcription of *hmsT* that encodes a protein containing GGDEF domain (Sun et al., 2012). Fur had no regulatory effect on *hmsHFRS*, which is responsible for the synthesis and translocation of biofilm exopolysaccharide across the cell envelope (Bobrov et al., 2008; Sun et al., 2012). Thus, Fur can either activate or repress biofilm formation based on genetic background and iron concentration.

Fur has been described as an iron-dependent transcriptional regulator. However, a few studies have also demonstrated Fur to regulate the transcription of some genes independent of iron (Davis et al., 2005; Mey et al., 2005). The data presented here demonstrate that Fur, independent of iron, represses the biofilm formation and c-di-GMP production by *V. cholerae*, as well as controls the transcription of *cdgD*, *vieSAB*, *vpsU*, and *vpsA-K*. Fur bound to the regulatory regions of *vieSAB* and *cdgD*/*vps* activates and represses their transcription, respectively. Fur sites for *vieSAB*, *cdgD*, *vpsA-K*, and *vpsU* were not identified by ChIP-seq analysis (Davies et al., 2011). The genes associated with Fur-binding sites obtained by ChIP-seq were also not identified in microarray studies (Davies et al., 2011). Discrepancies between the presented results and the results obtained by the ChIP-seq may be due to differences in experimental methods (ChIP-seq maps regulator binding sites *in vivo*), bacterial growth conditions, and/or bacterial genetic background.

Iron is an essential nutrient for life, but there is almost no free iron to use within the hosts during early infection (Ganz, 2018). However, the biofilms formed by *V. cholerae* strain C7258 seemed to be independent of iron concentration. It has been reported that early infections in humans may include some sort of biofilm formation (Faruque et al., 2006; Tamayo et al., 2010; Sengupta et al., 2016). A previous study demonstrated expression levels of Fur to be low during the adaptation period in LB broth at 37°C (Gao et al., 2018), suggesting that expression levels of Fur may also be low during the early infection stage. In addition, the QS master regulators AphA and HapR are highly expressed at low cell density and high cell density, respectively, with HapR also functioning at low cell density (Ball et al., 2017; Lu et al., 2018). AphA promotes biofilm formation, but HapR represses biofilm formation (Ball et al., 2017; Lu et al., 2018). Transcription of *fur* is directly repressed by HapR in *V. cholerae* (Gao et al., 2018). Therefore, during the early stage of infection, AphA may promote the biofilm formation by *V. cholerae*. Simultaneously and at least partly, the regulatory system composed of Fur and HapR inhibits the biofilm formation. In this case, *V. cholerae* appropriately forms biofilms, which may be conducive to the survival and pathogenicity of the pathogen.

The Fur site for *vieSAB* is located downstream of the transcription start site of *vieSAB* (Figure 5), and thus, this is an unusual binding position for a regulator to activate its target genes. The mechanism by which Fur directly activates the transcription of a target gene is complex with Fur binding sites usually located an approximate 100 bp upstream of the transcriptional start site of Fur-activated genes (Troxell and Hassan, 2013). Notably, the Fur binding site for *vieSAB* overlaps with the binding sites of H-NS (Figure 5), which has been demonstrated to be a repressor of *vieSAB* (Ayala et al., 2018). Thus, Fur may act as an anti-repressor of H-NS-mediated *vieSAB* repression in *V. cholerae* (Troxell and Hassan, 2013). The Fur site for each of the regulatory regions of *cdgD*, *vpsA-K*, and *vpsU* was found to be located far upstream of the core −35 element, which is unusual for transcriptional repression. It is possible that Fur can antagonize other transcriptional activators of *cdgD*, *vpsA-K*, and *vpsU*. For example, the Fur binding site for *vpsA-K* entirely overlaps with the binding site of VpsT, while that of *vpsU* is adjacent to the VpsR-box like sequence (Figure 5) (Ayala et al., 2015; Zamorano-Sanchez et al., 2015). Both VpsT and VpsR have been demonstrated to be expression activators of *vps* biosynthesis genes (Yildiz et al., 2001; Casper-Lindley and Yildiz, 2004). Moreover, the Fur binding site for *vpsA-K* also overlaps with the binding sites of H-NS (Figure 5), and thus Fur may cooperate with H-NS to repress *vpsA-K* transcription (Ayala et al., 2015, 2018). A similar mechanism has been demonstrated for the interaction of LeuO and H-NS with the *vieSAB* promoter in *V. cholerae* (Ayala et al., 2018). In addition, we observed that the predicted minimal Fur sites for *vpsA* and *cdgD* are located upstream of the detected Fur sites. This assertion is supported by our DNase I footprinting assay in the absence of Fe^2+^ and replaced by Mn^2+^. The DNA binding consensuses for Fur-Fe^2+^ and Fur-Mn^2+^ could be slightly different. In any case, the DNA-binding sites of Fur for target promoters cannot be simply predicted *in vitro* by the 19- or 21-bp palindromic sequences.

**FIGURE 6:**
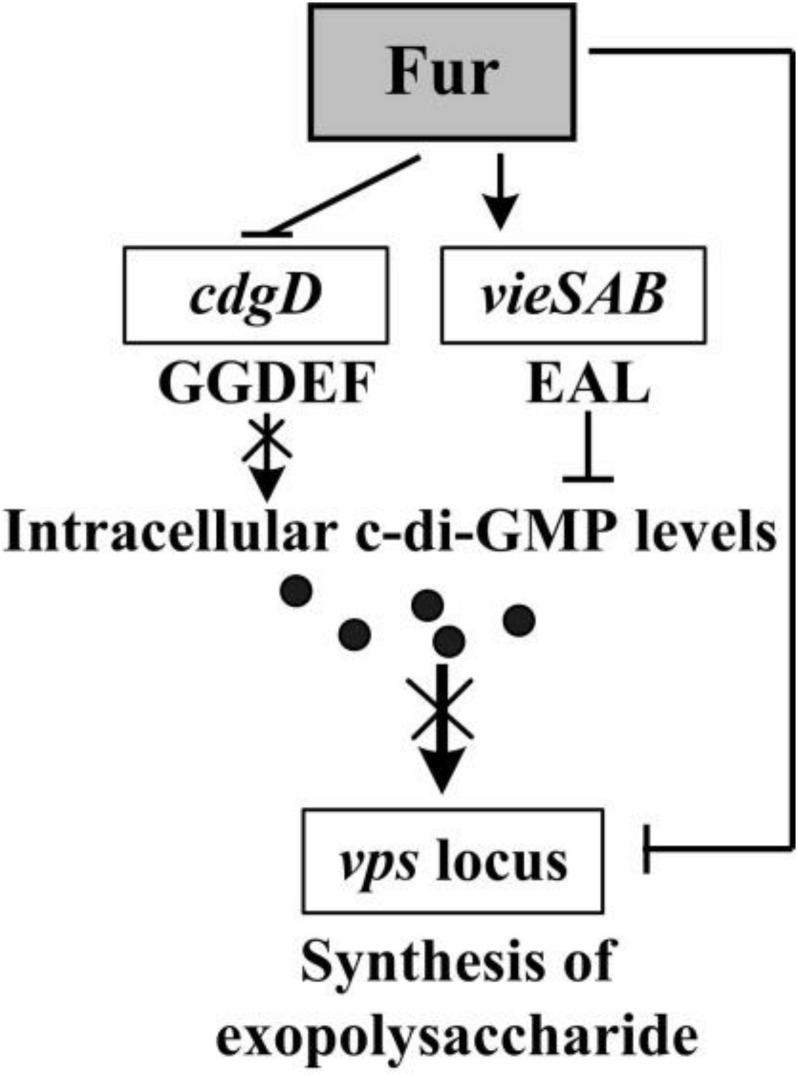
Regulatory circuit. The arrows and lines indicate positive regulation. The T-junctions indicate negative regulation. The regulatory details are described in the main text. The intersection lines indicated that the cellular pathways were disrupted. The black dots indicated c-di-GMP.

*cdgD* and *vieSAB* encode proteins with GGDEF and EAL domains (Tamayo et al., 2005; Syed et al., 2009; Dey et al., 2013), respectively. Therefore, repression of *cdgD* and activation of *vieSAB* by Fur may be responsible for the decreased intracellular c-di-GMP levels (Figure 2), and thus represses the biofilm formation in *V. cholerae*. H-NS represses biofilm formation of *V. cholerae* by directly acting on the promoter DNA regions of *vps* and *rbm* genes (Ayala et al., 2017). H-NS binding sites for these promoters overlap with the binding sites of VpsT (Ayala et al., 2015, 2017). At high c-di-GMP levels, H-NS is displaced by VpsT at these promoters, allowing expression of *vps* and *rbm* as well as biofilm production. At low levels of c-di-GMP, VpsT is displaced by H-NS, which then silences the expression of *vps* and *rbm* genes (Ayala et al., 2015). Thus, the decreased intracellular c-di-GMP levels caused by Fur may further result in a possibility that H-NS displaces VpsT from the promoters of *vps* and *rpm* genes to repress their transcription and the biofilm formation. However, no matter what mechanisms Fur adopts to repress the biofilm formation by *V. cholerae*, two different mechanisms may be concluded here from the data (Figure 6). First, Fur binds to and alters the transcriptional levels of *cdgD* and *vieSAB*, which may then decrease the intracellular levels of c-di-GMP. Second, Fur binds to promoter-proximal DNA regions of *vpsU* and *vpsA-K* to repress their transcription, which then decrease the production of exopolysaccharide. Taken together, a new physiological role for Fur, as a repressor of biofilm formation in *V. cholerae*, has been demonstrated.

## Data Availability Statement

The raw data supporting the conclusions of this article will be made available by the authors, without undue reservation.

## Author Contributions

HG, YZ, and BK conceived the study and designed experimental procedures and wrote the manuscript. LM, QQ, YQ, JZ, JLi, JLo, BD, HZ, and QS performed the experiments and carried out data analysis. All authors contributed to the article and approved the submitted version.

## Conflict of Interest

The authors declare that the research was conducted in the absence of any commercial or financial relationships that could be construed as a potential conflict of interest.

## Supplementary Material

Supplementary Figure 1 Cell density-dependent expression of Fur. The promoter-proximal DNA region of *fur* was cloned into the pBBRlux vector and transferred into WT to determine the luminescence activity under various OD_600_ values. The bacteria were cultivated at 30°C in LB broth with DP or iron supplementation.

Supplementary Figure 2 Transcription start sites for *cdgD*, *vpsA*, and *vpsU*. Negative numbers represent nucleotide positions upstream of the translation start site for each target gene. The transcription start sites are marked with asterisks and positions. The boxed sequences indicated the sequences of Adaptor.

Supplementary Table 1 Oligonucleotide primers used in this study.

Supplementary Table 2 Predicted Fur box-like sequences within target promoters.
