# Supplementary figures and images for: Fur Represses Vibrio cholerae Biofilm Formation via Direct Regulation of vieSAB, cdgD, vpsU, and vpsA-K Transcription
Source: Front Microbiol. 2020 Oct 22;11:587159. doi: 10.3389/fmicb.2020.587159 (PMC7641913; doi:10.3389/fmicb.2020.587159)

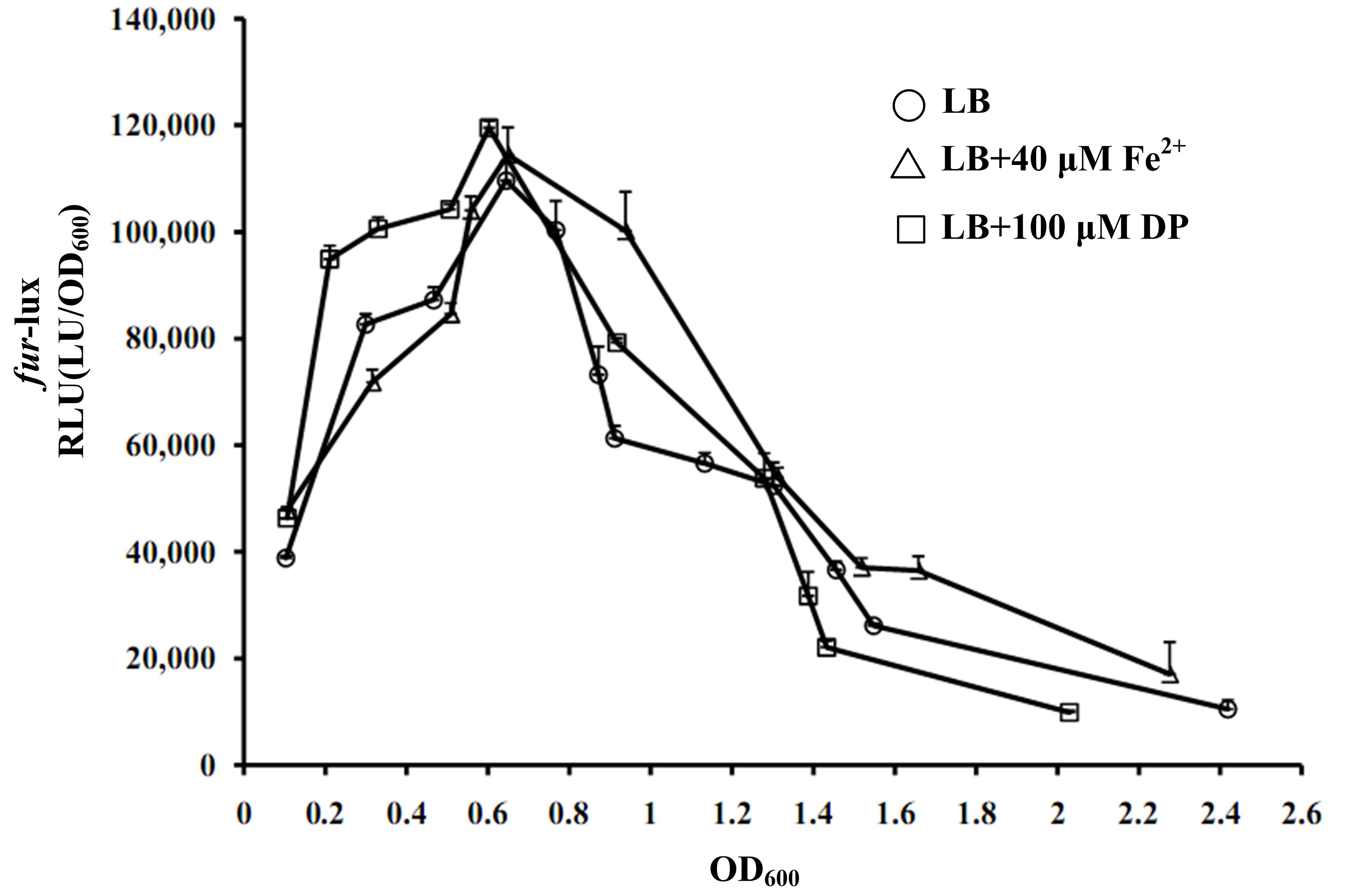

Supplement: Supplementary Figure 1 — Cell density-dependent expression of Fur. The promoter-proximal DNA region of fur was cloned into the pBBRlux vector and transferred into WT to determine the luminescence activity under various OD600 values. The bacteria were cultivated at 30°C in LB broth with DP or iron supplementation. [file Image_1.jpg]

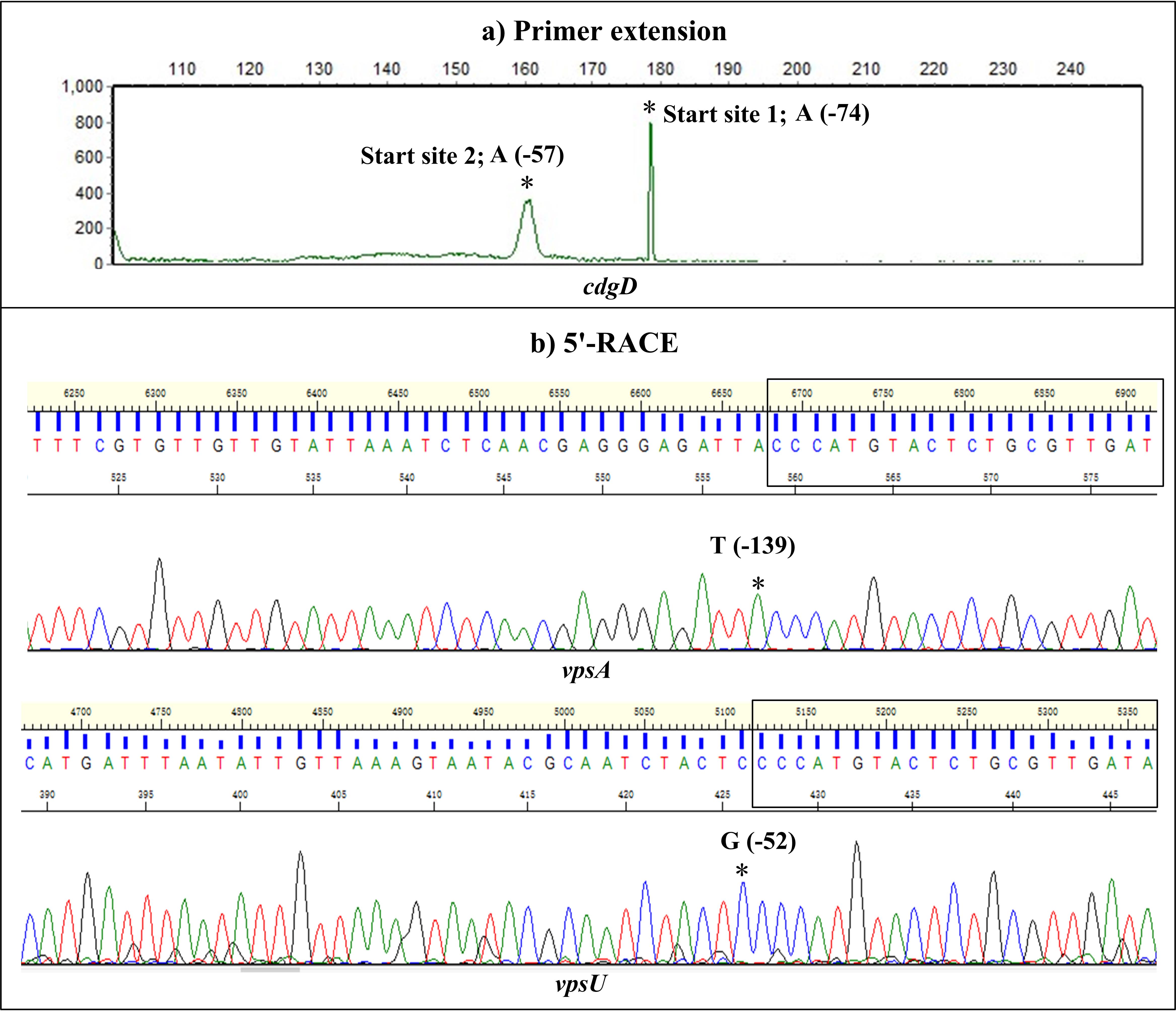

Supplement: Supplementary Figure 2 — Transcription start sites for cdgD, vpsA, and vpsU. Negative numbers represent nucleotide positions upstream of the translation start site for each target gene. The transcription start sites are marked with asterisks and positions. The boxed sequences indicated the sequences of Adaptor. [file Image_2.jpg]
