# Supplementary material for: Fur Represses Vibrio cholerae Biofilm Formation via Direct Regulation of vieSAB, cdgD, vpsU, and vpsA-K Transcription
Source: Front Microbiol. 2020 Oct 22;11:587159. doi: 10.3389/fmicb.2020.587159 (PMC7641913; doi:10.3389/fmicb.2020.587159)
Supplement: Supplementary Table 1 — Oligonucleotide primers used in this study. [file Data_Sheet_1.pdf]

Table S1. Oligonucleotide primers used in this study

Target	Primers (forward/reverse, 5'-3')
**Construction of mutants**	
fur	CGGGATCCTTCGTGTAAGGCAGCAGTAATC/CAGAGCGTAAAGCCTATGGATACTTTCCTGTTGATGTTC
	GAACATCAACAGGAAAGTATCCATAGGCTTTACGCTCTG/GGACTAGTAGATGAAGATGGTGTGGGAAAC
	CGGGATCCTTCGTGTAAGGCAGCAGTAATC/GGACTAGTAGATGAAGATGGTGTGGGAAAC
**Construction of complemented mutants**	
fur	GATTCTAGAAGGAGGAATTCACCATGTCAGACAATAACCAAG/GCGAAGCTTTTATTTCTTCGGCTTGTGAG
**Protein expression**	
fur	GCGGGATCCATGTCAGACAATAACCAAG/GCGAAGCTTTTATTTCTTCGGCTTGTGAG
**qPCR**	
vieS	AAGCAGCGAAAGCCAACT/CTTCAAATCCACGCAATAA
cdgD	GATTTACGATGGGCACCA/TTCACCGTCACGACAAGA
vpsA	TGATGCGTTGTTGGCTGTG/TGCTGCTCTGCGGTAGTG
vpsU	CACGCTAAAGACAGGAAA/TGCTCAGCAGGACAAATA
recA	AAGATTGGTGTGATGTTTGGTA/CACTTCTTCGCCTTCTTTGA
**Primer extension**	
cdgD	/AACCAAGTACAAACGCAAC-HEX

**Luminescence assay**	
vieS	GCGGAGCTCCGCCTAACGAGATGCTGT/GCGACTAGTCCTATCGGCTCCTGACCT
cdgD	GCGGAGCTCATCTCAGGTTTGGCTTCG/GCGGGATCCATGAGATTTCGCTTCCAG
vpsA	GCGGAGCTCCAATGGATAGGACCAATC/GCGGGATCCCCAAATACGATGAGAAC
vpsU	GCGACTAGTTGTATCGCTTGATTTCCG/GCGGGATCCACCCTAACTACGCACATG
**EMSA**	
vieS	GATGATTATGCACTCGATG/ATACCCTAACTACGCACATG
cdgD	TGGCTCTTTTTCTTAGCAAG/TTCGTCGTTATAAACAATC
vpsA	ATCTCAGGTTTGGCTTCG/AGCATGAAGTGTGCCTTTAC
vpsU	CTTCTGTAGGAATTTTCTCTC/CCTATCGGCTCCTGACCT
recA	AAGATTGGTGTGATGTTTGGTA/CACTTCTTCGCCTTCTTTGA
**DNase I footprinting**	
vieS	GTAAAACGACGGCCAGTCTTCTGTAGGAATTTTCTCTC/CAGGAAACAGCTATGACCCTATCGGCTCCTGACCT
cdgD	GTAAAACGACGGCCAGTATCTCAGGTTTGGCTTCG/CAGGAAACAGCTATGACAGCATGAAGTGTGCCTTTAC
vpsA	GTAAAACGACGGCCAGTTGGCTCTTTTTCTTAGCAAG/CAGGAAACAGCTATGACTTCGTCGTTATAAACAATC
vpsU	GTAAAACGACGGCCAGTGATGATTATGCACTCGATG/CAGGAAACAGCTATGACATACCCTAACTACGCACATG

M13	FAM-GTAAAACGACGGCCAGT/CAGGAAACAGCTATGAC-HEX
**5'-RACE**	
vpsA	/GGTGCAAAGTGGTACTGCGTGAGTGC
	/GTATCTGTAGGTGCAAAGTGGTACTG
vpsU	/CACTACTACGCCGATAAGGATCGG
	/GCCACCGCTCCAGATTCAATCAAC
UPM-Long	CTAATACGACTCACTATAGGGCAAGCAGTGGTATCAACGCAGAGT
UPM-Short	CTAATACGACTCACTATAGGGC

Table S2. Predicted Fur box-like sequences within target promoters

**Operon**	**First gene**	**Fur box-like sequence**		
		**Position&**	**Sequene**	**Score**
vieSAB	*vieS*	D-135…-117	AACGATATTTCTTTACATT	9.3
	cdgD	D-376…-358	CATGAGTATTTTTTTCATT	8.5
vpsA-K	*vpsA*	D-334…-316	AACGAAAATTTATCTCAAT	9.9
	vpsU	D-150…-132	AAATAAAACTACTTTCATT	7.2

&, ‘D’ indicates the direct sequence; minus numbers denote the nucleotide positions upstream of the translation start sites of indicated genes.
